# The relevance of context in understanding health literacy skills: Findings from a qualitative study

**DOI:** 10.1111/hex.12547

**Published:** 2017-04-12

**Authors:** Verna B. McKenna, Jane Sixsmith, Margaret M. Barry

**Affiliations:** ^1^ Health Promotion Research Centre Discipline of Health Promotion National University of Ireland Galway Ireland

**Keywords:** health experiences, health literacy, health‐care provider communication, qualitative

## Abstract

**Background:**

Conceptualizing health literacy as a relational concept, which involves how individuals interact with complex health and social systems, requires a greater understanding of the context of people's health experiences.

**Objectives:**

To describe individuals’ experiences of accessing, understanding, appraising and applying health information; explore the barriers and facilitators to using these skills; and to describe the experience of information exchange in health consultations.

**Design:**

A longitudinal qualitative methodology with thematic analysis of interviews was used. Health literacy levels were assessed using the HLS‐EU‐47–Item Questionnaire. Findings are presented from the first round of data collection.

**Setting and participants:**

Twenty‐six participants purposefully selected from a CVD risk reduction programme at three separate time points.

**Results:**

Four key themes identified: using health literacy capacities for managing health; psychological and structural factors that impact on these capacities; and the relationship quality with the health‐care provider (HCP). Although limited health literacy was prevalent across the sample (65%), all individuals were very proactive in attempting to utilize health literacy skills. Findings emphasize the importance of contextual factors such as the quality of communication with the health‐care provider, perceptions of control, attitudes to family medical history, navigating structural barriers and being supported in managing treatment and medication side‐effects.

**Discussion and Conclusion:**

Findings are relevant for health‐care providers in order to enhance the patient‐provider relationship and to ensure optimum health outcomes for all individuals regardless of health literacy levels.

## 1 INTRODUCTION

Health literacy concerns the capacities of people to meet the complex demands of health in modern society. It is viewed as an increasingly important component in the self‐management of illness and the ability to effectively engage in health promotion activities.^1^ While the research evidence consistently demonstrates poorer health outcomes associated with lower levels of health literacy, relatively little is known about how people develop their health literacy skills in the context of managing their health and illness, how this changes over time and the barriers and facilitators that may be experienced in this process.^1^, ^2^, ^3^, ^4^, ^5^, ^6^, ^7^

Although the importance of the social context of health decision making has been previously highlighted,^8^, ^9^, ^10^, ^11^, ^12^ conceptualizations of health literacy have been slow to move away from framing health literacy primarily as a capacity of the individual. The main emphasis of health literacy studies to date has been on measurement development and this has occurred with little associated research into health professionals’ communication skills.^13^, ^14^, ^15^ The majority of studies have focussed on the functional level of health literacy as outlined by Nutbeam^16^, ^17^ with far less work exploring the communicative and critical levels of health literacy.^9^ Chinn (2011) advocates the use of qualitative methodologies to explore “how people actually interact critically with health information in real‐life situations”.^18^ ^(p64)^

Lai et al. (2015) also argue that health literacy studies need to shift from a predominantly patient focus to one that encompasses health interactions and health contexts.^19^ The current study sets out to do this and employs a qualitative methodology, incorporating the HLS‐EU conceptual model,^15^ to explore individuals’ experiences. This is a recently developed comprehensive model of health literacy that emphasizes the capacities necessary to be considered health literate and to make decisions about health: access, understanding, appraisal and application, which can be linked to functional, interactive and critical levels of health literacy.^20^ The HLS‐EU model proposes that individuals who possess all four capacities are more likely to be able to successfully navigate three key dimensions of the health continuum: the health‐care setting, the disease prevention system and community‐based health promotion.^15^

This paper presents findings from the first phase of a larger longitudinal qualitative study, consisting of three phases, which aims to examine developments in the health literacy of individuals over time. The objectives of phase one were to: describe individuals’ experiences of accessing, understanding, appraising and applying health information; explore the context (ie the barriers and facilitators) to using these skills, and to describe the experience of information exchange in health consultations. The overall aim of this study was to explore the use of health literacy skills in the context of individuals managing risk factors for CVD.

## 2 METHODS

### 2.1 Study design

This study describes phase one of a longitudinal qualitative study design, which employs repeat interview methodology at three separate time points (see Table 1) to examine developments in health literacy of individuals over time. Data on health literacy levels were collected at time points one and three, and this contributed to a layered approach as advocated by Saldaña.^21^

**Table 1:** Overview of timeline and methods for overall longitudinal qualitative study

	Methods used		
Time points	Focus		
Phase 1: (Baseline: Beginning of programme)	To explore individuals’ experiences of using health literacy capacities in the management of health and illness.	Participants interviewed	HLS‐EU survey completed
Phase 2 (End of programme@12 wk)	To monitor developments and changes in the use of health literacy capacities.	Participants interviewed	
Phase 3 (1 yr follow‐up @ 12 mo)	To examine developments and changes in the use of health literacy capacities over time and to explore the barriers and facilitators in this process.	Participants interviewed	HLS‐EU survey completed

### 2.2 Participants

The concept of purposeful sampling is used in qualitative research to select individuals/sites for study because they can purposefully inform an understanding of the research problem and central phenomenon in the study.^22^In this study, purposeful sampling was employed to select individuals attending a community‐based structured cardiovascular risk reduction programme and so obtain the views and experiences of people with a range of risk factors for cardiovascular disease, as well as those with established disease (see Table 2). The twelve‐week programme integrates the care of individuals with established heart disease and those at high multifactorial risk of developing the disease, into a local community‐based programme^20^ that was originally developed at Imperial College London following the EUROACTION trial.^19^ The programme in this study is provided in a community setting. Initial recruitment took place in conjunction with the programme nurse who identified individuals who were cognitively able to participate and had an understanding of the English language. A unique feature of the programme is that partners of referred patients are also invited to complete the programme, and in this study, five partners were included. Recruitment took place between February and December 2014.

**Table 2:** Profile of study participants

Participants (n)	26
Gender (n, %)	
Male	10 (38%)
Female	16 (62%)
Age (mean, range)	59 (36‐76)
Education: highest level attained to date (n, %)	
Primary School (PS) level (Low)	3 (11.5%)
Incomplete PS (Low)	1 (3.8%)
Secondary intermediate level (Low)	7 (27%)
Completed secondary(Medium)	5 (19.2%)
Diploma/certificate(Medium)	5 (19.2%)
Primary degree (High)	1 (3.8%0
Postgraduate/higher degree (High)	4 (15.4%)
Social class^25^ (n, %)	
I (High)	1 (4%)
II (High)	7 (27%)
III (Medium)	1 (4%
IV (Medium)	3 (17%)
V (Low)	4 (4%)
VI (Low)	3 (17%
VII(Low)	7 (27%)
General health literacy level from HLS‐EU measure^23^ (n,%)	
Limited	17 (65)
Adequate	9 (35)
Health service access (n/%)	
Private health insurance	13 (50)
Medical card only	7 (27)
Private AND medical card	4 (15)
Public access only	2 (8)
Smoking	
% Current Smokers	12 (n=3)
Diet	
Mean Mediterranean Diet Score (optimal score≥9)	5
Physical Activity	
% NOT achieving targets (>5x/wk ≥30 min)	81 (n=21)
Anthropometrics	
%BMI≥25 kg/m^2^ (overweight)	27 (n=7)
%BMI≥30 kg/m^2^ (obese)	65 (n=17)
% Waist Circumference NOT at target	
Male≥94 cm & Females ≥80 cm	96 (n=25)
Blood pressure	
% BP NOT to target	
(>140/90 mm Hg for high‐risk individuals & >130/80 mm Hg for coronary/diabetes)	62 (n=16)
Cholesterol	
% Cholesterol NOT to target (TC >5 mmol/L & LDL > 3 mmol/L for high‐risk individuals and TC >4.5 mmol/L & LDL >2.5 mmol/L for coronary/diabetes)	42 (n=11)
% with other illness‐related risk factors (heart attack, stroke, Diabetes; Hypothyroidism, Psoriasis, Cardiac arrhythmia, heart disease (stents fitted), heart failure	73 (n=19)

### 2.3 Profile of study participants

All of the participants were commencing a twelve‐week CVD risk reduction programme and were referred through various pathways including general practice and hospital departments such as cardiology, stroke and endocrinology. Participant characteristics are summarized in Table 2.

### 2.4 Data collection procedures

#### 2.4.1 Interviews

Twenty‐six interviews, with an average length of 30 minutes duration were conducted. Semistructured interview guides were used to explore the development of health literacy and to identify changes in knowledge, attitudes and experiences over time (see Appendix S1). The development of the interview guide was informed by Sørensen's conceptual model of health literacy^15^ to explore all the capacities associated with health literacy. For phase one of the study, the interview guide focussed on the specific phase one study objectives. All Interviews took place at the community‐based programme building and were conducted by the first author (VMcK). Participants’ health literacy levels were assessed using the HLS‐EU‐47–Item Questionnaire^23^ to examine initial levels of health literacy. The use of this measure adds perspective to the qualitative data and allows for comparison with levels recorded for the overall Irish population.^20^ Interview and survey administration was piloted prior to commencement of data collection with a small number of individuals attending the structured programme.

### 2.5 Ethical considerations

The study was independently reviewed and approved by the Research Ethics Committee, National University of Ireland, Galway, in May 2013. All participants were provided with written and oral details of study participation and provided with written informed consent to participate in the study. Emphasis was placed on the voluntary nature of study participation, the removal of all identifiers and that all information would be anonymized.

### 2.6 Data analysis

#### 2.6.1 Interviews

All interviews were audio‐recorded digitally, transcribed verbatim and analysed using thematic analysis, which was facilitated through the use of N‐VIVO version 10 qualitative software. The study used a hybrid approach of inductive and deductive coding^24^ and theme development employing a thematic analysis methodology as advocated by Braun and Clarke (2006).

Credibility of findings was enhanced by returning to the original transcripts and through discussion with the other authors (MB and JS). A sample of transcripts was also read by MB, and initial codes and final themes were reviewed with both MB and JS. In addition, a sample of transcripts was independently coded by another experienced qualitative researcher.

#### 2.6.2 HLS‐EU measure

This measure^23^ was used primarily for descriptive purposes and to profile the participants further. The instrument is scored for four indices: a general health literacy index (reported in this paper) and three dimension‐specific indices (health care, disease prevention and health promotion—these will be addressed in the longitudinal study findings). The raw scores are categorized to denote the following levels of health literacy: inadequate, problematic, sufficient and excellent.^1^, ^15^ These were further combined to yield scores for limited and adequate levels of heath literacy. The HLS‐EU has previously been validated,^23^ and good internal reliability was demonstrated in this study (General α=0.91; Health Care α=0.80; Disease Prevention α=0.80; Health Promotion α=0.89). The measure was administered by the first author (VMcK) in face‐to‐face meetings with participants.

## 3 RESULTS

General health literacy scores were calculated for all participants and indicated a high level of limited health literacy across the sample at 65% (n=17) with a lesser proportion having an adequate level of health literacy (35%, n=9). The limited level of health literacy reported here is significantly higher than levels reported in the overall European Health Literacy survey^1^ (47%) and in the Irish sample of the European survey (40%).^20^ These findings correspond with those for population subgroups with lower education and social class levels, and higher rates of disease and health service use.^1^In this study, the sample was mixed across demographics as depicted in Table 3 below. Statistical tests for differences in general health literacy scores for education and social class^25^ were not significant (*P=*.265 and .389, respectively).

**Table 3:** Mean health literacy scores across education and social class

*Health literacy level from HLS‐EU measure* ^*23*^	*Limited (*n*=17;* $\overset{¯}{x}$ *=29)*			*Adequate (*n*=9;* $\overset{¯}{x}$ *=34)*		
*Education level*	Low	Med	High	Low	Med	High
	n=8;$\overset{¯}{x}$=28	n=6; $\overset{¯}{x}$=32	n=3; $\overset{¯}{x}$=29	n=3; $\overset{¯}{x}$=36	n=4; $\overset{¯}{x}$=39	n=2; $\overset{¯}{x}$=41
*Social class*	Low	Med	High	Low	Med	High
	n=7; $\overset{¯}{x}$=29	n=7; $\overset{¯}{x}$=29	n=3; $\overset{¯}{x}$=30	n=4; $\overset{¯}{x}$=36	n=1; $\overset{¯}{x}$=35	n=4; $\overset{¯}{x}$=41

### 3.1 Interview data

The interviews yielded rich data relating to participants’ experiences and strategies for accessing, understanding, appraising and applying health information across various health contexts. The data also identified barriers and facilitators which can impact on health literacy practices for the individuals, and these are depicted in the four interlinked themes set out in Table 4 below. The findings are presented in terms of these four themes as well as relevant subthemes and categories.

**Table 4:** Themes and additional participant quotations

Participant number P1,P2, P3…P26	Gender M: Male F: Female	Health literacy level A: Adequate L: Limited	Example quote label: P1FL
Theme	Subtheme	Categories	Examples of additional participant quotes
Using health literacy capacities for self‐management of health and illness	Health information seeking	Keeping motivated Active and passive information seeking Appraising information Making sense of information	So I would read anything. And if they give you anything in the hospital any time I have been in, I will keep it, and I will maybe read it when I come home, but I would read it again a week later, do you know? (P23FL)
			Well sometimes the information, if you're not into the terms that the doctors in the hospital use it's just like you have to go looking up about this, say different words and then you're wondering.. And then you spend ages looking that up… So it's kind of like, it's alright if you know the medical terms of everything; then if you don't you're like just, it's kind of like trying to learn a new language. (P1FL)
	Side‐effects of medication use	Decisions about treatments Concerns re QoL	I did mention it a couple of times and she kind of said “oh you're better off to stay on it’ but I don't know to be honest. I feel it nearly makes you too passive or too, you know, you just, as I say, just let everything flow by you kind of thing. (P5FL)
Psychological factors that impact use of health literacy capacities	Perceptions of control	Being confident and proactive Not having control Dealing with family history	And as my own brother died with it… from a major heart attack, I would have you know more of an interest. And I have another brother that got a stroke two years later and they were only in their fifties. (P23FL)
	Emotional reactions	Anxiety and coping	Feeling that I could get into the car and drive and stay in somewhere like X or somewhere. I have a longing to say go to X and have a few days and I'm afraid to do it and that's not living, that's my big issue, yeah, that's terrible… I haven't met anybody, friends have said we'd meet in X and stay the night, I'm afraid to do the trip, I'm afraid to be away from home (P21FL)
Structural factors that impact use of health literacy capacities	Being able to access health services	Health service access Having/not having health insurance Waiting lists Fragmentation of services	It might be not the right word but I feel worthless and useless and demoralised. To think that if you haven't got the money your health is screwed; just ridiculous. It should be, in a perfect world, waiting lists shouldn't be three or four years long. Nothing I can do about it.(P11FL)
	Environment	Living environment (rural, urban) Affordability	I'm on my own, I'm separated, I only work September till May, and so kind of from January to May you are saving to get the few extra bob for the summer – so, no, I can't afford private health, and I have no money to pay for it (P26FL)
Quality of relationship with the HCP	Qualities of the HCP	Listening, good rapport, trust, feeling cared for	Oh it's easy to talk to them, yeah. And I've a good GP like, and he'll refer me in, and I'll meet up with the doctor and ask all the questions you want (P17ML)
			If something like that came up that I thought that maybe I shouldn't be using, I would ask him, you know, that I would take his, his word would be the most important to me (P23ML)
			And he knows that I'm, as he calls it, highly idiosyncratic, that I'd be allergic to medication, and stuff, and everything. So he's very good at trying to find one that will work for me, you know, and he knows and I know that if I try it for a month, whether it's going to work or not (P26FL)
	Accessing and appraising information with the HCP	Positive and negative experiences Seeking referrals Active and passive relationships	Like I suffer with HS, I can never pronounce it correctly, Hidradenitis suppurativa, it's just abscesses and boils, all the time I suffer with and not many GPs really know a lot about it. I asked him to refer me to a dermatologist and he said “what the hell do you want to see a dermatologist for?” I said because I need to see a dermatologist. But “oh, you're looking at those sites again.” And I said but you can't give me the information I need and I would like to see a dermatologist. “Ok, I'll refer you.”(P11FL)
	Communicating	Positive experiences Negative experiences	He talks normal talk rather than doctor [talk] and I'm not being disrespectful.He goes down to your own level and that's what I like about him. Like there would be a name for a tablet there and it could be, you name it, forty letters long but he brings it down to a simple, do you know what I mean? And that kind of thing, once that's explained I know exactly where I'm going. (P13MA)

### 3.2 Using health literacy capacities for self‐management of health and illness

#### 3.2.1 Health information seeking

Participants in this study were managing a wide range of CVD risk factors as well as managing chronic illness, and they varied in the detail of information they wished to know about their conditions. Some were very proactive in seeking out detailed information from different sources while others preferred to view their doctor as the sole point of access to health information:

I wouldn't be somebody that'd be going home and researching what the doctors are telling me. I just take them at their word. (P14MA)

In responding to ill health, participants demonstrated efforts to apply actions to prevent disease and promote health and highlighted the challenges of maintaining motivation to sustain those activities. Participants emphasized the gap between *knowing* and *doing* which was an on‐going struggle for all participants. This included those who had established cardiac conditions and those undertaking more general lifestyle changes:

I find that awful hard; that's the hardest part. Motivation is incredibly difficult for me. (P3ML)

Participants used a variety of means to access health information including the Internet, newspapers, radio, medical leaflets, doctor and pharmacist. For those who use the Internet, *Google* was the predominant method to search for information. Others combined accessing information with an appraisal process using multiple sites, making comparisons and bringing information back to the GP for further clarification. Participants expressed caution in over‐reliance on the Internet as the sole source of health information:

I know if I'd anything seriously wrong I'd definitely check with the GP. I wouldn't fully rely on the internet at all. (P6FL)

However, it was also viewed as a useful way to supplement understanding of information received from the doctor:

You can go to a doctor… but to be able to go home then, Google it up, print it off, read it and study it, and then go back with some of the formative questions, is good. (P26FL)

In terms of appraising health information, participants who regularly used the Internet to access health information were able to differentiate between sites which are generally deemed to be trustworthy (medical sites) and other sites. Participants also stated that they preferred using medical sites over those which are predominantly based on patient experiences (such as patient forums).

..there are some good sites there and some bad ones. So you have to, weed out the chaff from the wheat as it were. The Mayo clinic‐they really explain everything spot on. (P3ML)

Attempts to understand health information can be impeded by unclear instructions as depicted in the quote below where a participant describes the confusion she felt where there was conflicting information provided on a medical leaflet describing the timing of medication dosage for a colonoscopy procedure:

I was in a real panic because I'd read this and it said you must not eat or drink anything from midnight the night before the test and then I read this leaflet and it said second dose at seven am the next day. (P8FL)

#### 3.2.2 Side‐effects of medication use

Participants with both limited and adequate levels of health literacy engaged in self‐questioning regarding the side‐effects of various medications, and it clearly influenced their decisions on whether or not to take up or continue with various treatment plans:

I'd be afraid like, I mean all my blood tests that I get taken they're fine, they're perfect and if I were to go taking statins well who knows what'd happen?(P12FL)

These concerns also impacted on decisions to change medication dosages or to cease taking medicines altogether:

I was wondering about, was it wise for me to be taking all this poison? I would refer to it as. I just went off the tablets. (P7MA)

Quality‐of‐life considerations were important in making decisions about treatment plans/medication use:

So if I feel that the downside of a medication has the potential to cause me serious problems in another area, then I would ask for an alternative. (P19FA)

### 3.3 Psychological factors that impact use of health literacy capacities

#### 3.3.1 Perceptions of control

Participants described feelings of control and confidence in relation to managing their health, which could impact on how proactive or passive they were in activities related to using health literacy capacities. Perceptions of control were linked to how individuals engaged with health information/health services. For some, being in control was important and is related to personal responsibility for health:

I don't accept what people will tell me about my health unless I'm happy about it myself …no offence to the medical profession but they're not going to take the time to figure out all the nooks and crannies of my medical issues. So I'm responsible for that myself. (P2FA)

This is linked to an awareness of personal characteristics needed to be proactive:

I would be somebody who'd make the phone call, keep asking the questions until I find the right person to speak to. (P2FA)

For others, a sense of limited control pervaded their attitudes to managing health issues. Having an awareness of a family history in relation to illness or risk factors and how one copes with that also has a bearing on the control individuals felt in relation to their health. Some participants referred to not having any choice but to get on and make behaviour/lifestyle changes; in some cases, this was equated with ‘*doing what one is told’*(PR06). Some participants reported having little control over their health situation. Some dealt with family history by attempting to disengage from it:

I thought I could do whatever I wanted; I was perfect. I did know in the back of my mind about my mother but you know you kind of shrug it off…But I didn't look sick or anything like that. (P7MA)

For others, an awareness of risk factors rooted in family history acted as an impetus to gain more knowledge about the condition:

Find out more about diabetes really and heart disease because it's something that's big in our family…so that I'll know to take care of myself and my family. (P1FL)

A number of participants described situations where they experienced long delays in getting a diagnosis despite their on‐going health concerns and continued interactions with their doctor. These experiences were linked to feeling quite powerless in relation to trying to manage health concerns when there was no clear diagnosis:

I kind of blame myself because for ten years I thought I was complaining of a kidney infection and when it turned out to be ovarian problems, that was a bit of a concern that it hadn't been picked up. (P8FL)

#### 3.3.2 Emotional reactions

Psychological aspects associated with diagnoses and on‐going treatment of illnesses are important influencing factors that can hinder the application of health literacy skills and the active engagement of individuals regardless of levels of health literacy. These factors include the anxiety and shock of coping with diagnoses, which can also impact on the control one feels in relation to health issues:

The technician up in the x Clinic, he said have you been offered a CRTD? [Cardiac resynchronization therapy] and I said I have but I'm scared of it and I don't want the idea of something shocking me. (PR21FL)

Difficulties in understanding information were sometimes linked to emotional barriers such as dealing with the shock following a diagnosis. Participants had devised strategies to overcome these barriers such as having another person in attendance and bringing notes and/or questions into the consultation. One participant described how the doctor's understanding of her anxieties and the involvement of a family member facilitated the communication process to ensure that the health information was understood correctly:

When I was in my consultant's and she said to my husband I'm going to tell you now because she won't believe me… I wouldn't believe. I would go home with the worst scenario possible. (P9FA)

Similarly, another participant described how the shock of her husband's stroke diagnosis impacted on her ability to process the information:

He tries to explain to us…but…, when he got the stroke, some of it went over my head. (PP23FL)

Another participant who had previously worked in the health service highlighted her realization of the difficulties of taking in and understanding information when one is unwell:

I'm sure there's bits I forget and taking in information when you're sick, I never realized how different it is. It's frightening….I just gave people out their pills and did not have a clue what they were going through. (P21FL)

### 3.4 Structural factors that impact use of health literacy capacities

In discussing their health management experiences, participants identified a number of factors at the health‐care system and broader community and environmental levels that influenced their capacities to access, understand, appraise and effectively utilize health information.

#### 3.4.1 Being able to access health services

Within the Irish Health Service, some individuals with private health insurance have more timely access to consultants, diagnostic and treatment services compared to those without health insurance (public patients). Participants without health insurance described difficulties accessing needed health services. In the Republic of Ireland, primary health‐care services are not free at point of access except for those holding a medical card. Some participants had experience of both public and private health services use and were able to reflect and compare experiences. Those with private health insurance highlighted the timely access to services and equated this with earlier diagnosis:

And I know that I wouldn't be seen to, when I had that irregular heartbeat, that I'd be put on a waiting list and I mightn't get a good consultant then… that's why I got such good service because I had insurance. (P20FA)

Some participants found that the limited integration of services can make the management of multiple appointments/health‐care interactions difficult to manage:

There's nobody looking at all the whole file – you go in, they look at their little bit, they ask you the same questions you were asked before…there is no continuity at all through the hospitals. (P26FL)

Participants who do not have health insurance highlighted the issue of long waiting lists to see consultants as well as the poor coordination of medical appointments across different geographic locations:

I was referred to the pain clinic by Mr X maybe three years ago as an emergency and I'm still waiting. Two weeks ago they phoned me up to offer me an appointment in x but it'll be on‐going appointments so I'm not travelling to x because at the moment I'm travelling to y with other health problems.(P11FL)

#### 3.4.2 Environment

Participants identified how their living environments (including community and working) could facilitate or impede engagement with health practices. Rural dwelling participants highlighted the positive aspects of rural living (fresh air, wide open spaces, safety for children) while also identifying challenging issues (no footpaths, reduced safety for walking and cycling, limited access to groceries nearby).

I suppose in the countryside it's harder to stick with stuff and be more active apart from walking and stuff like that because there's nothing there; have to drive everywhere compared to in towns. Even with the shopping … there's not that much choice in the countryside. (P1FL)

A number of participants who had retired form work noted that it was much easier to engage in lifestyle changes outside of the work environment.

It's a different environment actually…when I was working I was on the road a good bit …it would have forced me into a car rather than doing healthier forms of transport you know. (P16ML)

#### 3.4.3 Affordability

Financial considerations were perceived as barriers or facilitators to being able to engage with health activities. Some participants (all with higher levels of health literacy) described themselves as “*fortunate enough”* and “*privileged”* to be able to afford

All the things that promote wellbeing…they all cost money…you have to be able to afford to do it, you know. (P19FA)

### 3.5 Quality of relationship with the HCP

#### 3.5.1 Qualities of the HCP

The relationship with the HCP (mainly GP) and the quality of that relationship emerged as central in using health literacy skills. Characteristics of the HCP relationship could act as a barrier or facilitator to information seeking and appraisal as well as to the overall interaction and communication experiences of participants. Trust, being listened to, having a good rapport and feeling comfortable and cared for were all important factors identified:

It's so important for somebody to listen to you and for them to understand what the problem is and how the patient is coping. (P12FL)

The perception of *caring* is clearly important, and this is particularly relevant for those who are managing serious illness:

When I was told that my heart function was so low I got so scared and Dr X phoned me several times…to see how I was, I'd never had calls from a cardiologist to know how I was, which was lovely. (PR21FL)

#### 3.5.2 Accessing and appraising information with the HCP

Many participants were involved in seeking and obtaining health information such as looking up specific information, asking questions of the doctor, having a strategy to keep information and putting the information to use. Many of these activities are also an important component of the appraisal process whereby the HCP is directly involved. Participants varied in their level of engagement with active information seeking. For some, information was obtained by attending talks (for example, through the diabetes clinic) and noting relevant information as it came up. Others were much more proactive in seeking out different sources of information, engaging and using the information in consultation with the GP. A common part of the appraisal process was bringing information found elsewhere into the consultation, and some participants had experienced negative reactions to this:

So I did a bit of research on the internet and I found that this particular drug, X, if it does give you a cough it means there's something wrong with your heart and I actually said that and she said “ah, you're reading too much.” (PR21FL)

Some participants, with both limited and adequate health literacy levels, had actively sought out a referral from their GP. One participant linked her insistence on a referral for a mammogram to the early detection of breast cancer:

So I went to my GP and she examined me, now I know she couldn't feel anything, so she wasn't going to send me to anywhere. But I said “I want to go for a mammogram”. So only for that I wouldn't have gone, and I had it. (P18FL)

Another participant used her knowledge of the difficulties of detecting an underactive thyroid using blood tests to insist on a referral:

Most thyroid readings come out as normal, but you can still have a lot of the symptoms of an underactive thyroid, which I have. So I insisted that my GP refer me to a consultant, and I was referred, and I am now on medication for my thyroid. (P19FA)

Despite the overall high level of limited health literacy in the study sample, the majority of participants were active in seeking out health information. However, a small number described more passive interactions with health‐care providers and engagement with health information:

I would never ask the doctor. If he says it to me that's ok like you know. I'm not a kind of a guy now that would be asking or looking for answers. If it comes, it comes, do you know what I mean? (PR10ML)

Some participants described negative experiences in their interactions with HCPS that correspond with a more paternalistic model of communication:

Well we do talk but sometimes I do feel that maybe it's kind of like, you know, “what would you know, this is my area.” I'm not a confrontational person so I wouldn't like to kind of rock the boat. (P8FL)

## 4 DISCUSSION

The overall aim of this study was to explore the use of health literacy skills in the context of individuals managing risk factors for CVD. The findings of this study are consistent with a perspective of health literacy as a relational concept which emphasizes individuals’ interactions with complex health and social systems.^26^, ^27^ The findings have generated a number of important insights into contextual factors influencing how health literacy capacities may be used. These are relevant for health‐care providers in order to enhance the patient‐provider relationship and to ensure optimum health outcomes for all individuals regardless of health literacy levels.

Study findings have highlighted that the health‐care provider (HCP), and most often the General Practitioner (GP), is central to the process of navigating health information and is generally seen as the most trustworthy source of information, even where the Internet is routinely used. However, some participants had also experienced more negative interactions where they felt that their opinion was not respected and this acted as an impairment to their relationship with the HCP which in turn may act as a barrier in using health literacy capacities. A positive response by the health‐care provider to information brought to consultations is recognized as an important factor in creating positive patient‐provider relationships.^28^ Study findings indicate that the communication style of the HCP can either facilitate or act as a barrier to information exchange, and other studies have highlighted how this can subsequently contribute to empowerment or disempowerment of the individual.^29^ Timely access to appropriate health services was another potential source of disempowerment identified in this study. Long waiting lists, highlighted in this study, can act as a barrier for individuals to engage with health issues and the timely uptake of treatment plans. Where people are managing multiple conditions, the lack of a coherent service can be problematic. Health literacy skills may have limited impact in instances where structural barriers to health service access exist. Typically, those with more limited health literacy are going to experience greater challenges in this situation and are also more likely to be impacted adversely by social demographic factors.^30^

The issue of empowerment is important to consider and is understood here to refer to psychological empowerment that includes the constructs of personal control, a proactive approach to life and a critical understanding of the socio‐political environment.^31^ It entails being able and motivated to bring about changes not only in personal behaviour but also in the social situations and the organisations that influence one's lives.^32^

The exact nature of the relationship between health literacy and empowerment continues to be contested in the literature. However, there is a growing consensus that while health literacy does not automatically lead to empowerment, it may well be understood as an instrument in the process.^33^ Both concepts are regarded as distinct but closely connected through knowledge, skills and power dimensions.^33^, ^34^, ^35^ Porr et al. (2006) further consider that individuals’ competencies, self‐efficacy, critical thinking and reflection are important factors in the process.^35^ Sykes et al. (2013) used a concept analysis approach to identify critical health literacy as being similar to empowerment, the key attributes of which were health knowledge, confidence, self‐efficacy and empowerment.

Feelings of being in control or having limited control are central to how health literacy capacities may be utilized in managing health. Study findings highlight that being in control is associated with feelings of greater confidence which in turn can contribute to more proactive engagement with health issues. Barriers to control identified include those at the personal or psychological level and those at the broader social and structural levels. The psychological aspects of managing illness can impact on abilities to access, understand, appraise and use health information. In this study, participants described how information given during times of stress may be difficult to take on board and understand. In addition, individuals may refuse treatments altogether based on their fears associated with the treatment. Health‐care providers need to have an understanding of the contexts of such fears and be able to offer support in a caring way to allay these fears. Having regular reviews to discuss the implications of long‐term treatments routinely built into consultations, and providing individuals with information on making changes to medications/treatment plans as appropriate, is important in this regard. Such an approach corresponds with a model of shared decision making where doctors and patients jointly participate in a treatment decision‐making process and come to some negotiation of which treatment is chosen and implemented.^36^

Although participants in this study demonstrated the ability to be quite proactive in their interactions with HCPs, it cannot be assumed that all individuals have the skills to be proactive regarding issues such as requesting referrals or second opinions and/or questioning medication use. A routine and consistent approach to raising such issues in the consultation by the HCP could be beneficial in terms of improving adherence and health outcomes. Initiation of such discussions by the HCP could also mitigate concerns over time‐pressured consultations that may impact on individual's confidence to bring up issues. Another important element is the importance of having a clear understanding of how individuals interpret and understand their risk in relation to family history, which can be particularly relevant in the management of CVD risk factors. Familial risk models indicate that persons will attempt to either cope with or control disease vulnerability (Walter and Emory, 2005). Findings in this study identified that some individuals needed to attempt to exert control over familial risk factors, and this may be linked to beliefs about the contribution of nature and nurture to disease.^37^

An important finding relates to individuals’ concerns regarding the side‐effects of treatments. Greater knowledge on the part of HCPs of the health beliefs and attitudes of the patient will help to ensure that appropriate health information is provided. For example, treatments could be better tailored where there is knowledge about the patient's attitude to medication usage and more lifestyle‐focussed treatment plans could be incorporated where feasible. It is important that HCPs are aware of the extent of such concerns and the context for them in order to support the individuals to make appropriate care decisions. Lower levels of health literacy can affect key decision‐making outcomes,^38^ and the use of formal decision aids may be useful.^39^, ^40^ Without adequate support in this process, some individuals may have adverse health outcomes, for example, if they cease taking medications associated with particular side‐effects.

### 4.1 Strengths and limitations of the study:

One strength of this study is that it combines data on health literacy levels with the qualitative experiences of individuals. Individuals with limited health literacy are well represented in the study. The participants in the study were sampled from those at the beginning stages of a community‐based CVD risk reduction programme and so differs from the health‐care‐based patient samples used in other studies.

The study design could have been strengthened using a stratified purposeful sample which would allow for a clear differentiation between groups with high/low health literacy. However, this was not possible due to the nature of recruitment (rolling intake) into the community‐based programme. Ultimately, study participation relied on the voluntary participation of individuals and so it is possible that those individuals who were most engaged with health issues were more likely to take part.

## 5 CONCLUSION

Participants in this study, with varied levels of health literacy, are striving “to make informed choices, reduce health risks and improve quality of life”.^41^ Individuals interacting with HCPs all have different levels of health literacy which in turn can impact on how health information is accessed, understood, appraised and applied. HCPs need to have an increased awareness and understanding of these contexts. There were no clear patterns regarding health literacy levels and experiences discerned in this study in relation to either education or social class levels. However, what does seem to be most important are contextual factors such as the quality of communication with the health‐care provider, perceptions of control, attitudes to family medical history, navigating structural barriers and being supported in managing treatment and medication side‐effects. Capacity at the critical level of health literacy entails moving from an understanding of basic health information to being able to contextualize it and to use this to gain control and/or change the determinants of a particular outcome. Further research involving follow‐up with participants as part of the larger on‐going longitudinal study will offer increased insights into barriers and facilitators to developments in health literacy over time.
