# Supplementary material for: The relevance of context in understanding health literacy skills: Findings from a qualitative study
Source: Health Expect. 2017 Apr 12;20(5):1049–60. doi: 10.1111/hex.12547 (PMC5600250; doi:10.1111/hex.12547)
Supplement: Supplementary file 1 [file HEX-20-1049-s001.doc]

**Longitudinal study: Phase 1 interview questions**

**Q1: Can you tell me about what has brought you to this programme?**

[probe as relevant regarding various health issues]

**Q2: Tell me about the kind of things that you do to keep yourself healthy?**

[probe physical and mental health; how easy/difficult is this?]

**Q3: How do you usually get information about *your* health or health issues in general?**

[probe regarding healthcare; disease prevention; health promotion. Prompt: Can you think of a recent example where you have had to find out something in relation to health?]

**Q4: How easy or difficult would you generally find it to understand any health information that you get?**

[Prompt Can you give me an example of a time when you found a piece of health information easy/or difficult to understand?]

**Q5: Tell me about how you work out what information is useful for your own situation?**

[prompt Can you give me an example of how you’ve done this in the past?]

**Q6: Tell me about the kind of things that you do with the health information you have obtained?**

**Q7: Can you think back to a visit, in the last 12 months or so, to your doctor (GP) or perhaps as an outpatient appointment at a hospital , how did you interact/communicate with your health care provider?**

[Prompts: did you have chance to ask questions/ Do you generally feel comfortable asking questions?]

**Q8: In general, tell me about whether you feel that you are in a position to easily make changes that you think could benefit your health.**

[Probe: What makes it easy?/What makes it difficult /]

**Q9: Can you tell me a bit about your local community such as how involved you are in your local community?**

[prompt do you think that where you live can affect your health and well-being, why/why not and how? ]

**Q10 What changes in yourself do you hope will result from this experience ? (programme participation)**
